# HEvOD: A database of hurricane evacuation orders in the United States

**DOI:** 10.1038/s41597-024-03100-x

**Published:** 2024-03-05

**Authors:** Harsh Anand, Negin Alemazkoor, Majid Shafiee-Jood

**Affiliations:** 1 Department of Systems and Information Engineering, University of Virginia, Charlottesville, 22903 USA; 2 Department of Civil and Environmental Engineering, University of Virginia, Charlottesville, 22903 USA

**Keywords:** Natural hazards, Policy

## Abstract

Assessing and improving the effectiveness of evacuation orders is critical to improving hurricane emergency response, particularly as the frequency of hurricanes increases in the United States. However, our understanding of causal relationships between evacuation orders and evacuation decision-making is still limited, in large part due to the lack of standardized, high-temporal-resolution data on historical evacuation orders. To overcome this gap, we developed the Hurricane Evacuation Order Database (HEvOD) – a comprehensive database of hurricane evacuation orders issued in the United States between 2014 and 2022. The database features evacuation orders that were systematically retrieved and compiled from a wide range of resources and includes information on order type, announcement time, effective time, and evacuation area. The rich collection of attributes and the resolution of the data in the database will allow researchers to systematically investigate the impact of evacuation orders, as a vital public policy instrument, and can serve as an important resource to identify gaps in current policies, leading to more effective policy design in response to hurricanes.

## Background & Summary

Hurricanes devastate communities and have long-lasting impacts in coastal, low-lying areas. In the United States, hurricanes have caused the most deaths and destruction among all recorded weather disasters^1,2^. As the frequency and severity of hurricanes rise in the United States, so does the urgency to improve emergency preparedness and response in hurricane-prone areas. Evacuation is an integral component of hurricane emergency response which can play a critical role in reducing the damage and loss of life during hurricanes^3–6^. Therefore, understanding evacuation decision-making is critical to improving hurricane emergency response.

While the decision to evacuate in response to an approaching hurricane is complex and multifaceted, evacuation orders are widely considered one of the influential factors^6–13^. Yet, our understanding of causal relationships between evacuation orders (and their different attributes) and evacuation decision-making is still limited. This is mainly because studies typically use a case study approach and rely on post-event surveys and interviews. Although these studies have provided significant and valuable insights, they are not well suited for policy analysis and design due to limited generalizability and hypothetical bias^8,14–19^; this significantly inhibits our ability to systematically evaluate the effectiveness of evacuation orders as a public policy instrument. The prevalence of geotagged social media data and high-fidelity mobility data in the past few years has enabled researchers to, at least partially, overcome hypothetical and sampling biases^20,21^. Nevertheless, generalizing the findings beyond limited geography and conducting causal learning and reasoning are still limited due to the lack of a comprehensive, standardized, and high-temporal-resolution record of historical evacuation orders. Compiling such a database is, however, challenging and requires navigating through various complexities. Evacuation laws and policies vary widely across states^22^. Specifically, states differ on who has the legal authority to order mass evacuations (e.g., local jurisdictions, governor). State and local entities also differ in how they communicate the details of evacuation orders (e.g., order type, evacuation area). Additionally, a wide range of platforms are typically used to disseminate the orders to the public (e.g., press conferences, press releases), and these announcements are typically widely disseminated using public and social media, all making the process of data collection and verification challenging and time-consuming.

In this paper, we introduce the **H**urricane **Ev**acuation **O**rder **D**atabase (*HEvOD*) – a novel database of evacuation orders issued by official state and local agencies in response to hurricanes that impacted the United States between 2014 and 2022. The database features a high-temporal resolution archive of evacuation orders collected from multiple sources, including official websites and social media accounts of local and state governments and government agencies, as well as news platforms. Corresponding to each evacuation order, the database includes information on the type of the order (mandatory or voluntary), the announcement date and time of the order, the effective date and time of the order (where applicable), and the areas that were the target of the order for evacuation. The database also includes information regarding the State of Emergency as declared by the governors in response to the events.

The rich collection of attributes and the high-temporal resolution of the data compiled in the database allow researchers to systematically investigate the impact of evacuation policies, providing a unique opportunity to address a wide range of research and policy questions related to hurricane evacuation planning and decision-making. For example, coupling evacuation orders with high-resolution location-specific mobility data will allow researchers to study the effectiveness of evacuation orders. Furthermore, the spatial component of the database (i.e., areas subject to evacuation orders) will allow researchers to assess the effectiveness of evacuation zoning and zone-based evacuation to draw insights into ways to improve the communication of evacuation orders, particularly in areas with lower-than-expected compliance rates. Finally, the database can be used in studies that aim to derive generalizable conclusions by focusing on multiple hurricanes over time and across various geographical locations. Therefore, *HEvOD* will fill a significant gap in existing datasets and serve as an important resource to reveal gaps in current policies and practices, leading to more strategic and effective evacuation planning and disaster mitigation during hurricanes.

## Methods

**Fig. 1:**
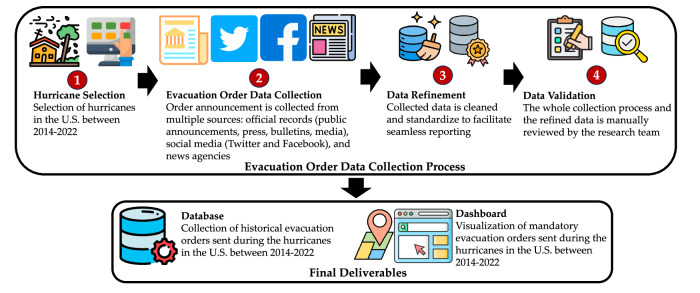
Summary of compiling the *HEvOD*. The procedure involved (1) selecting hurricanes from past events, (2) gathering related evacuation orders from various sources, (3) refining and standardizing the collected data, and, (4) having the entire data reviewed and verified by the research team.

Figure 1 provides a schematic overview of the process of compiling *HEvOD*, which comprises four major steps: (1) hurricane selection, (2) data collection, (3) data refinement, and (4) data validation. We will demonstrate the first three steps in this section and describe the last step as well as the limitations of our methodology in the ‘Technical Validation’ section. A thorough discussion of the final deliverables is presented in the ‘Data Records’ section.

### Hurricane selection

**Fig. 2:**
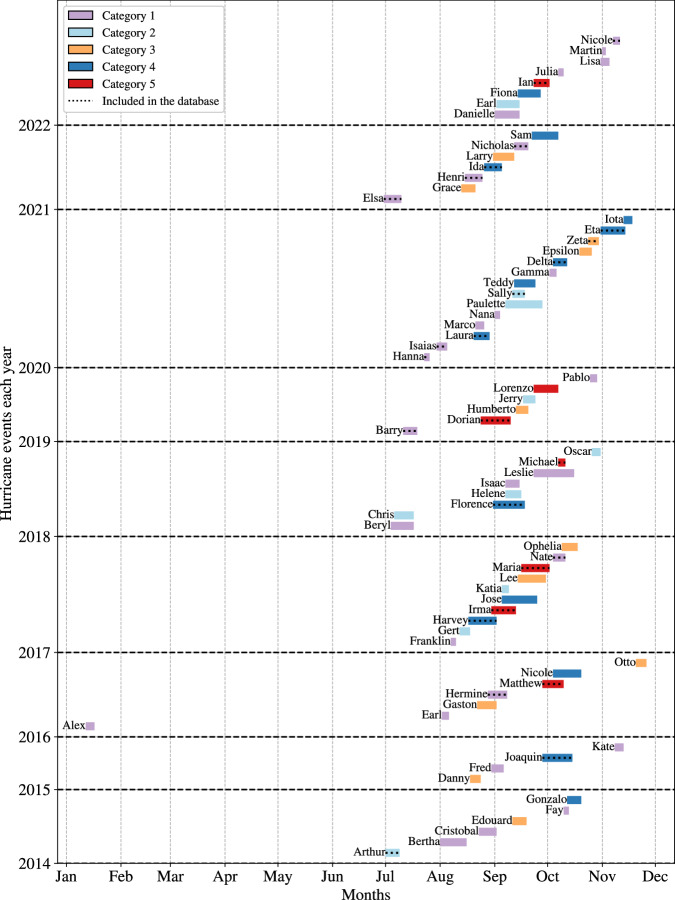
Chronological timeline of Atlantic hurricanes from 2014 to 2022. The colors indicate the peak category (based on the Saffir-Simpson hurricane wind scale) the hurricanes reached. The 25 hurricanes that are considered in the database are highlighted. Note that we excluded Hurricane Jose (2017) and Hurricane Marco (2020) due to their significant overlaps with Hurricane Irma and Hurricane Laura, respectively.

The hurricanes included in *HEvOD* were systematically selected from the National Hurricane Center’s archive of tropical cyclones^23^. Specifically, of the 161 tropical cyclones that formed in the Atlantic Ocean between 2014 and 2022, we first selected 70 events that were classified as hurricane, i.e., tropical cyclones with maximum sustained winds of 74 mph (64 knots) or higher, as shown in Fig. 2. We then selected a subset of these hurricanes – specifically 27 hurricanes – that either made a direct landfall or approached the United States close enough to pose a significant threat, specifically those events that prompted the declaration of at least a State of Emergency due to their potential or realized impacts. We then excluded Hurricane Jose (2017, Category 4) and Hurricane Marco (2020, Category 1) from the database because they significantly overlapped with Hurricane Irma (2017, Category 5) and Hurricane Laura (2020, Category 4), respectively, making it challenging and confusing to separate orders. Consequently, *HEvOD* includes 25 hurricanes. Table 1 provides more details about these hurricanes, including those states that declared a State of Emergency, the hurricane category (referring to the peak category reached by the hurricane) as defined by the Saffir-Simpson Hurricane Wind Scale^24^, and the dates the hurricanes formed and dissipated.

**Table 1:** List of hurricanes included in *HEvOD*.

Year	Event Name	Peak Category	Formed Date	Dissipated Date	States with SoE* Declaration
2014	Hurricane Arthur	Category 2	7/1/14	7/9/14	North Carolina
2015	Hurricane Joaquin	Category 4	9/28/15	10/15/15	Maryland, North Carolina, New Jersey, South Carolina, Virginia, Delaware
2016	Hurricane Hermine	Category 1	8/28/16	9/8/16	Florida, Georgia, North Carolina, Maryland, New Jersey, Virginia
2016	Hurricane Matthew	Category 5	9/28/16	10/10/16	Florida, Georgia, South Carolina
2017	Hurricane Harvey	Category 4	8/17/17	9/2/17	Texas, Louisiana
2017	Hurricane Irma	Category 5	8/30/17	9/13/17	Florida, Georgia, South Carolina
2017	Hurricane Maria	Category 5	9/16/17	10/2/17	Florida, North Carolina
2017	Hurricane Nate	Category 1	10/4/17	10/11/17	Alabama, Louisiana, Florida, Mississippi
2018	Hurricane Florence	Category 4	8/31/18	9/18/18	North Carolina, South Carolina, Virginia
2018	Hurricane Michael	Category 5	10/7/18	10/11/18	Alabama, Florida, Georgia, South Carolina, North Carolina, Virginia
2019	Hurricane Barry	Category 1	7/11/19	7/19/19	Louisiana, Mississippi
2019	Hurricane Dorian	Category 5	8/24/19	9/10/19	Florida, South Carolina, North Carolina, Virginia, Georgia
2020	Hurricane Hanna	Category 1	7/23/20	7/26/20	Texas
2020	Hurricane Isaias	Category 1	7/30/20	8/5/20	Florida, North Carolina, Virginia
2020	Hurricane Laura	Category 4	8/20/20	8/29/20	Texas, Florida, Louisiana
2020	Hurricane Sally	Category 2	9/11/20	9/18/20	Mississippi, Louisiana, Alabama, Florida
2020	Hurricane Delta	Category 4	10/4/20	10/12/20	Louisiana, Alabama
2020	Hurricane Zeta	Category 3	10/24/20	10/30/20	Louisiana, Alabama, Mississippi
2020	Hurricane Eta	Category 4	10/31/20	11/14/20	Florida
2021	Hurricane Elsa	Category 1	6/30/21	7/10/21	Florida, Georgia
2021	Hurricane Henri	Category 1	8/15/21	8/25/21	Connecticut, New York, Rhode Island
2021	Hurricane Ida	Category 4	8/26/21	9/5/21	Mississippi, Louisiana
2021	Hurricane Nicholas	Category 1	9/12/21	9/20/21	Texas, Louisiana
2022	Hurricane Ian	Category 5	9/23/22	10/2/22	Florida, South Carolina, North Carolina, Virginia
2022	Hurricane Nicole	Category 1	11/7/22	11/11/22	Florida

*State of Emergency.

### Data collection

The data included in *HEvOD* were collected and curated from multiple sources. Given that emergency and warning messages are widely shared and spread during natural disasters, including hurricanes, the data were primarily sourced from official announcements issued by the local and state governments or government agencies to ensure reliability and accuracy. These official announcements are typically issued and communicated to the public in the form of public announcements, bulletins, press releases, and press conferences. To retrieve these official announcements, we first compiled a list of websites, and Facebook and Twitter accounts for the state and local governments as well as the emergency management agencies in states that are frequently hit by hurricanes, including Florida, Louisiana, Virginia, North Carolina, South Carolina, and Texas. For these states, we manually searched the official websites for hurricane-related bulletins, news updates, press releases, and official advisories during the hurricane activity period. For states other than those listed above, guided by the actual path and projected trajectory of hurricanes, we manually conducted an open web search to collect the State of Emergency declarations and evacuation orders from official sources.

Recognizing the critical role of social media in disseminating information during natural disasters, many government and government agencies use their official social media accounts (specifically, Twitter and Facebook) to share major announcements with the public. Therefore, we supplemented the data retrieval process by searching the official Twitter and Facebook accounts of state and local government agencies. For Twitter, we leveraged both direct and open search approaches. The direct search involved the use of predetermined handles and the term ‘evacuation,’ accompanied by the region and hurricane timeline. For states whose official Twitter handles have not been compiled, we deployed open searches with the term “evacuation” and the hurricane timeline. In addition to manually searching, we used Python3 and the Twitter API v2, designed for academic research, to download tweets from 2014 to 2022. Filtering through keyword-based regular expressions allowed us to isolate relevant tweets, which were then manually cross-verified against online reports and articles. For Facebook, because the advanced search option is limited, we manually identified the relevant posts by going through the account timeline during the period of hurricane activity. Finally, because news media outlets also play a critical role in disseminating warning messages during hurricanes, we utilized online news media platforms to fill the gaps that we observed during the data retrieval from official sources, ensuring comprehensive coverage of hurricane-related information. The ‘Data Records’ section provides additional information regarding the specifics of the database records, including how the announcements were extracted from multiple sources and added to the database.

### Data refinement

Data refinement and standardization were essential parts of creating *HEvOD*. During the data collection process, we organized the data in a tabular format for each hurricane. However, as we began integrating the information from multiple hurricanes into a unified, coherent format, we noted some discrepancies that required attention to ensure consistency across the database. For example, during Hurricane Matthew in 2016, South Carolina’s governor did not issue traditional mandatory or voluntary orders. Instead, she stated, “We don’t do voluntary or mandatory anymore. An evacuation is an evacuation^25^.” Similar issue was observed in Florida’s Escambia County during Hurricane Nate in 2017, where the terms ‘voluntary’ or ‘mandatory’ were not used in relation to evacuations^26^. We also encountered data related to curfew timings rather than specific evacuation orders during Hurricane Florence in 2018 in several South Carolina counties^27^. Given these disparities, we established a few rules for normalization. In cases where the order type is not explicitly specified or broader terms such as ‘evacuation’ are used, as was the case during Hurricane Eta in 2020^28^, it is classified as a mandatory evacuation order. Terms such as ‘Voluntary/Phased evacuation order’ or ‘Recommended voluntary evacuation order’ were all classified as voluntary evacuation orders. These standardization measures ensured a uniform and coherent structure within our database, making it an orderly source for evacuation order information.

## Data Records

*HEvOD* is stored in and publicly available from LibraData^29^, the University of Virginia’s Scholarly Research Dataverse. Additionally, we provide an online dashboard to visualize the mandatory evacuation orders issued during each hurricane. The dashboard is available at http://www.hurrevacorder.info/ where users can also access the entire database and execute queries to download the data.

**Fig. 3:**
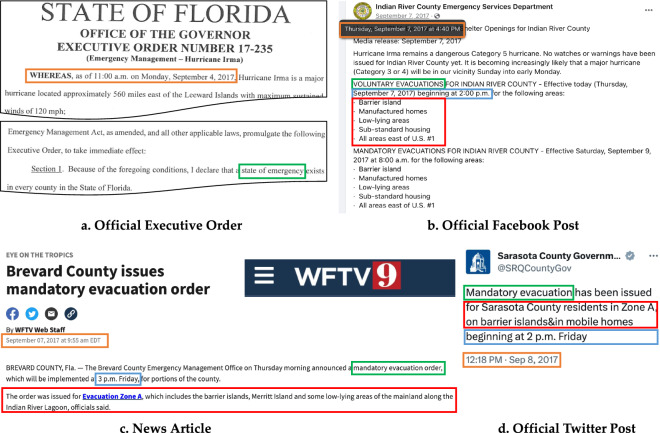
An example of how different attributes of the database (Table 2) were extracted from different sources for the case of Hurricane Irma (2017, Category 5). The sources used in this example include: (**a**) government postings, (**b**) Facebook posts, (**c**) news articles, and (**d**) Twitter posts. The specifics of the order announcement are highlighted in the orange box; the green box denotes the type of evacuation order or declaration; the red box marks the regions impacted by the order; and the effective date of the order is contained within the blue box. The organized record of these values in *HEvOD* can be seen in Table 3.

*HEvOD* presents a standardized database of historical evacuation orders, as well as State of Emergency declarations associated with the 25 hurricanes that impacted the United States between 2014 and 2022. The database is compiled in the form of a spreadsheet. Utilizing a systematic naming convention, “Year Name”, each tab within the file corresponds to a specific hurricane event. For the State of Emergency declaration, which appears in the database under the “Order Type” column, the main attributes are the time and date of the announcement. For the evacuation orders, there are four main attributes: first is the order type, which can be mandatory or voluntary; second is the announcement time, i.e., when the authorities first issue the evacuation order; third is the effective time, i.e., when the evacuation order officially goes into effect, which mainly applies to mandatory evacuation orders; and, fourth is the spatial element of an order which designates the high-risk areas that are subject to evacuation. Some states, such as Florida, have implemented a zoning system and use *evacuation zones* to communicate the orders, whereas some other states like Louisiana employ a more localized approach, using well-known landmarks or highways to delineate the evacuation areas. Table 2 provides a complete list of attributes in the database. We use Hurricane Irma (2017, Category 5) as an example and demonstrate in Fig. 3 how the main data attributes of the database were extracted from multiple sources. Once extracted, these attributes are then added to the database, as shown in Table 3. Along with the spreadsheet, we provide a single comma-separated values (CSV) file combining the orders and the State of Emergency declarations associated with all 25 hurricanes. The attributes excluded from the CSV file include ‘Day of the Week’, ‘Comments’, ‘Sources Type’, ‘Last Accessed’, and ‘Sources’. Together with our dataset, we also provide simple scripts in Python and R to read data from the CVS file.

**Table 2:** A list of columns in *HEvOD* with their respective definitions.

Column Name	Description
Event Name	The hurricane name.
Order Type	Includes “mandatory” and “voluntary” evacuation orders; may also include “State of Emergency”. A State of Emergency is declared by the governor when a disaster has occurred or is imminent that is severe enough to require State aid to supplement local resources in preventing or alleviating damages, loss, hardship, or suffering. Evacuation orders are protective actions used in certain emergencies to help save the lives of residents and first responders. A mandatory evacuation order means residents should leave their homes immediately, whereas a voluntary evacuation means residents can choose to stay at home but heading somewhere safer is highly recommended. If residents choose to ignore a mandatory evacuation order, they are fully responsible for their safety because police, fire, and emergency medical services are suspended in areas where a mandatory evacuation order is in place once the order goes into effect.
Order Type Code	Numerical codes for ‘Order Type’. ‘0’ for a State of Emergency, ‘1’ for a Mandatory Evacuation Order, ‘2’ for a Voluntary Evacuation Order, and ‘3’ signify the lifting of Mandatory and/or Voluntary Evacuation Orders.
Announcement Date	The date when the order was issued.
Day of the Week	The day of the week corresponding to the date the order was issued.
Announcement Time	The time when the order was issued.
Time Zone	The time zone applicable to the ‘Announcement Time’.
State	The state in which the order was issued.
County	The county (or parish in the case of Louisiana) in which the order was issued. Does not apply to the State of Emergency.
County FIPS	Five-digit Federal Information Processing Standard code to uniquely represent ‘County’ (where applicable).
Evacuation Area	The area that is the target of the evacuation order. Does not apply to the State of Emergency.
Effective Date	The date when the evacuation order goes into effect (where available). Does not apply to the State of Emergency.
Effective Time	The time when the evacuation order goes into effect (where available). Does not apply to the State of Emergency.
Comments	Section for any pertinent supplementary information (if any).
Source Type	Category of sources used to record the order details.
Last Accessed	The date when the referenced sources were last accessed.
Sources*	URL to the sources used to document the details of the order.

The example values for each column can be seen in Table 3.

*The line item may contain multiple sources, each included individually in separate columns.

**Table 3:** Samples records (for illustration purposes) of *HEvOD* for Hurricane Irma (2017, Category 5) corresponding to sources in Fig. 3.

Column Name	Line Item 1	Line Item 2	Line Item 3	Line Item 4
Event Name	Hurricane Irma	Hurricane Irma	Hurricane Irma	Hurricane Irma
Order Type	State of emergency	Voluntary evacuation order	Mandatory evacuation order	Mandatory evacuation order
Order Type Code	0	2	1	1
Announcement Date	9/4/17	9/7/17	9/7/17	9/8/17
Day of the week	Monday	Thursday	Thursday	Friday
Announcement Time	11:00:00	16:30:00	10:00:00	12:20:00
Time Zone	EDT	EDT	EDT	EDT
State	FL	FL	FL	FL
County	All counties	Indian River County	Brevard County	Sarasota County
County FIPS		12061	12009	12115
Evacuation Area		Barrier island, manufactured homes, low-lying areas, substandard housing, all areas east of US	Zone A, which includes barrier islands, merritt islands, and low-lying areas along the Indian River Lagoon	Resident in Zone A, barrier island, mobile homes
Effective Date		9/7/17	9/8/17	9/8/17
Effective Time		14:00:00	15:00:00	14:00:00
Comments				
Sources Type	Official executive order	Official Facebook posting	News posting	Official Twitter posting
Last Accessed	12/23/23	12/23/23	12/23/23	12/23/23
Source 1	Link to official report	Link to Facebook posting	Link to news	Link to Twitter posting
Source 2			Link to Facebook posting	

## Technical Validation

The database went through multiple stages of quality assessment and verification. The research team first conducted an internal review of the data collection and extraction process. This included compiling a comprehensive list of sources while ensuring their credibility and reliability. Once finalized, the data entries for each hurricane were populated and cross-checked. The database includes a direct link to the source materials, as well as the date when the source was accessed and the information was retrieved for full transparency. The entries were then reviewed again on a hurricane-by-hurricane basis to ensure data quality and accuracy. In this round of review, each evacuation order entry was subjected to a detailed comparison against secondary sources, such as articles, reports, and websites, to verify the data retrieved from primary sources and ensure completeness. In particular, news agencies provided an additional layer of validation, verifying data compiled from official government sources and supplementing information derived from social media posts. We also searched for official post-hurricane reports and were able to find only one report related to Hurricane Irma (2017, Category 5)^30^ that provides statistics on the total number of counties under mandatory evacuation orders. *HEvOD* recorded mandatory evacuation orders in 42 counties on September 11, which closely aligns with the report’s findings of 39. The methodology employed by the report to compute the daily evacuation numbers is unclear and is assumed to be cumulative. Therefore, our extensive multi-step review process has minimized the likelihood that erroneous records are included in the database, reinforcing its utility and reliability in hurricane-related studies.

However, it is important to highlight the limitations of our database. First, while we attempted to consistently retrieve and collect the announcement date and time associated with each evacuation order, there are 17 orders (all voluntary except one) across Hurricanes Nate, Dorian, Elsa, and Ian that have missing records. Second, given the nature of voluntary evacuation orders, officials typically do not report the effective time associated with these orders. But even in case of mandatory evacuation orders, the effective time is not always reported. Therefore, when available, we include the effective date and time; otherwise, the entries remain blank to preserve data integrity and credibility. Finally, while our methodology to retrieve, collect, and verify the data has been designed to maximize reliability and accuracy of the data, we acknowledge the possibility of missing records or inconsistencies in our database due to lack of standardized reporting, the retrospective nature of the process, or our oversight.

## Example Analysis

**Fig. 4:**
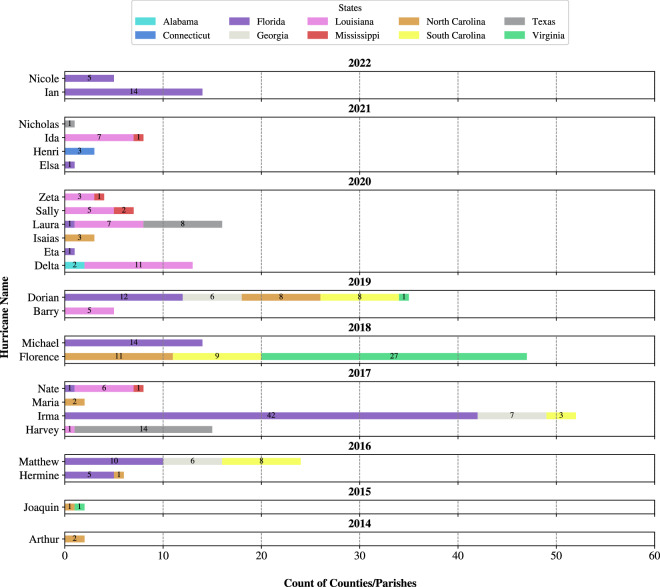
Number of counties that issued mandatory evacuation orders for the hurricanes considered in the database between 2014 and 2022.

**Fig. 5:**
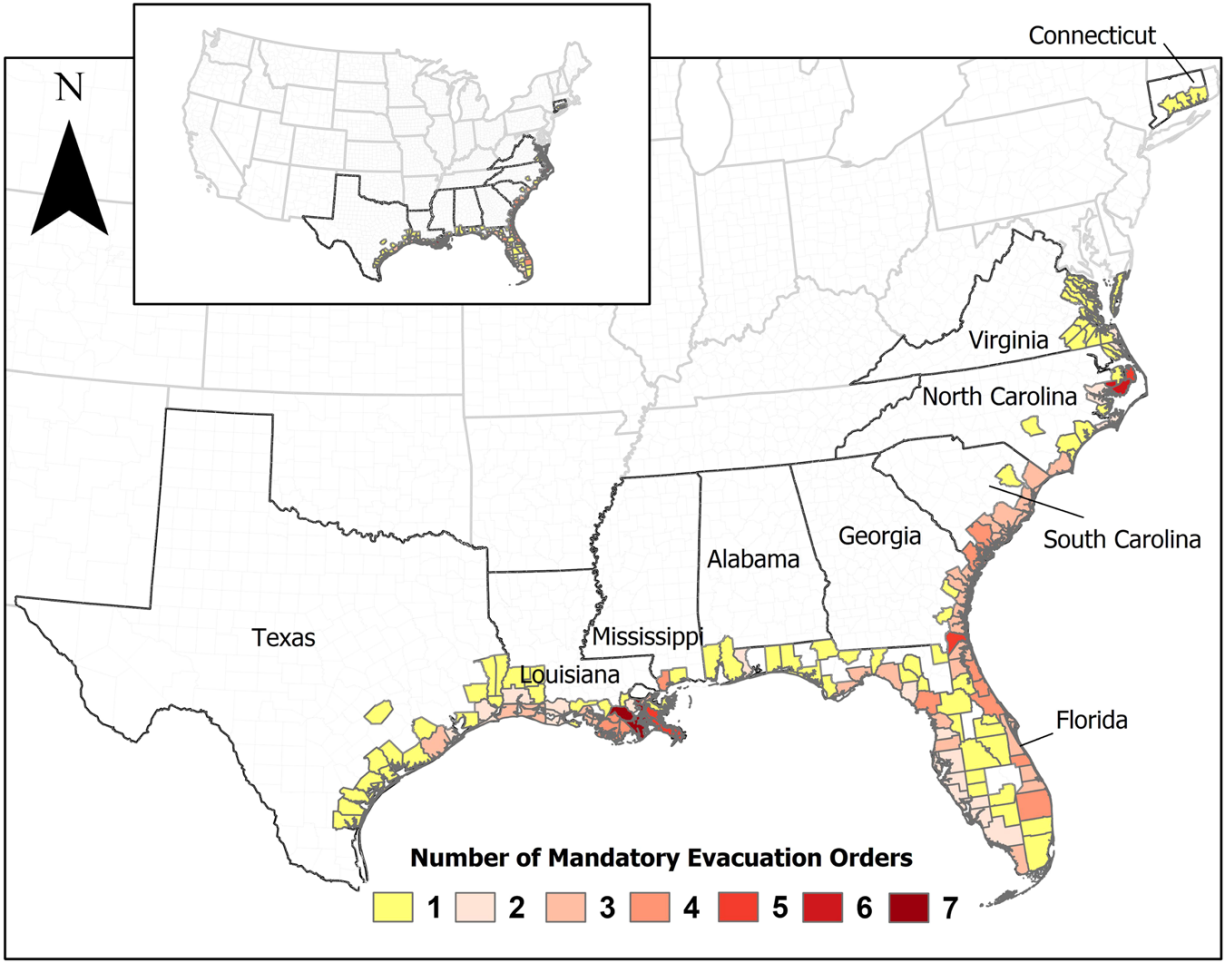
The total number of mandatory evacuation orders issued by each county for the hurricanes considered in the database between 2014 and 2022.

A summary of mandatory evacuation orders issued for the hurricanes that impacted the United States between 2014 and 2022 compiled from *HEvOD* is shown in Fig. 4. The figure shows that Hurricanes Florence, Dorian, Irma, and Matthew led to the most mandatory evacuation orders. The figure also shows that around 50% of the mandatory evacuation orders in this period were issued in Florida and Louisiana alone. Figure 5 shows the spatial distribution of these orders and highlights that a total number of seventeen counties, located in Mississippi, Louisiana, Florida, South Carolina, and North Carolina, issued at least four mandatory evacuation orders in response to different hurricanes in the span of 2014–2022.

## Data Availability

Tweets were extracted from Twitter using publicly available scripts and the ‘Twitter API v2 for academic research’. No other code was used in the development of the database.
